# Microplastics Exposure Causes Negligible Effects on the Oxidative Response Enzymes Glutathione Reductase and Peroxidase in the Oligochaete *Tubifex tubifex*

**DOI:** 10.3390/toxics8010014

**Published:** 2020-02-15

**Authors:** Costanza Scopetani, Maranda Esterhuizen, Alessandra Cincinelli, Stephan Pflugmacher

**Affiliations:** 1 Faculty of Biological and Environmental Sciences Ecosystems and Environment Research Programme, University of Helsinki, Niemenkatu 73, Lahti FI-15140, Finland; maranda.esterhuizen-londt@helsinki.fi (M.E.); stephan.pflugmacher@helsinki.fi (S.P.); 2 Korea Institute of Science & Technology (KIST Europe) Environmental Safety Group. Joint Laboratory of Applied Ecotoxicology Campus E 7.1 66123 Saarbrücken, Germany; 3 Helsinki Institute of Sustainability (HELSUS), Fabianinkatu 33, 00014 Helsinki, Finland; 4 Department of Chemistry “Ugo Schiff”, University of Florence, Sesto Fiorentino, 50019 Florence, Italy; alessandra.cincinelli@unifi.it; 5 Department of Chemistry “Ugo Schiff”, University of Florence, and Consorzio Interuniversitario per lo Sviluppo dei Sistemi a Grande Interfase (CSGI), Sesto Fiorentino, 50019 Florence, Italy

**Keywords:** microplastic, polyethylene, *Tubifex tubifex*, aquatic oligochetes, mortality, oxidative stress, glutathione reductase, peroxidase, microplastic exposure, freshwater environments

## Abstract

Microplastics (MPs) are emerging pollutants, which are considered ubiquitous in aquatic ecosystems. The effects of MPs on aquatic biota are still poorly understood, and consequently, there is a need to understand the impacts that MPs may pose to organisms. In the present study, *Tubifex tubifex,* a freshwater oligochaete commonly used as a bioindicator of the aquatic environment, was exposed to fluorescent polyethylene microspheres (up to 10 µm in size) to test whether the oxidative stress status was affected. The mortality rate of *T. tubifex*, as well as the activities of the oxidative stress status biomarker enzymes glutathione reductase and peroxidase, were assessed. In terms of oxidative stress, no significant differences between the exposure organisms and the corresponding controls were detected. Even though the data suggest that polyethylene MPs and the selected concentrations did not pose a critical risk to *T. tubifex*, the previously reported tolerance of *T. tubifex* to environmental pollution should be taken into account and thus MPs as aquatic pollutants could still represent a threat to more sensitive oligochetes.

## 1. Introduction

Plastic pollution is one of the primary environmental concerns we are facing today [1]. Microplastics (MPs), typically referred to as pieces smaller than 5 mm in any dimension [2], have been found on beaches, in oceans, seas, rivers, and lakes [2,3,4,5,6,7,8]. One of the biggest concerns regarding MP pollution is that marine and freshwater biota can mistake MP particles for food. MPs can be ingested by benthic and pelagic organisms belonging to different trophic levels [9], including mussels [10], lugworms [11], amphipods [12], zooplankton [13], and fish [14]. Some studies showed that with ingestion possible internal damages and blockages [9,15] may occur. Ingested MPs can act as vectors for transferring chemicals, additives, and other persistent organic compounds (such as Polybrominated diphenyl ethers (PBDEs) and Polychlorinated biphenyls (PCBs)) adsorbed from surrounding waters to biota [16,17,18,19]. However, the effects of MPs on exposed biota have not been extensively investigated, and the physiological effects remain poorly understood. Thus, there is a current need to gather data to deepen our understanding of their impacts. A few studies have demonstrated that MPs could induce oxidative stress in the organisms able to ingest them [20,21]. Browne et al. [11] studied the effect of MPs on the oxidative status of lugworms (*Arenicola marina*) demonstrating that animals exposed to polyvinyl chloride (PVC) microparticles were more susceptible to oxidative damage by up to 30%. Jeong et al. [20] showed that in response to microplastic-induced reactive oxygen species (ROS), antioxidant enzymes such as glutathione reductase (GR), glutathione peroxidase (GPx), glutathione-S-transferase (GST), and superoxide dismutase (SOD) were activated in the monogonont rotifer (*Brachionus koreanus*). The authors exposed the monogonont rotifer to three different polystyrene MP sizes (0.05, 0.5, and 6 µm) and observed that the toxicity was size-dependent, and the smaller the particles, the more toxic. More recently, Lu et al. [21] showed that the freshwater fish *Danio rerio* (zebrafish) exhibited a higher oxidative stress status after seven days of exposure to polystyrene MP, revealed by increased SOD and catalase (CAT) activities. Deng et al. [22] tested the effect of fluorescent polystyrene MP in mice, revealing increased activities for SOD and GPx and decreased CAT activity, signifying the potential health risk MPs represent to biota. Nevertheless, only a few of these studies report on the physiological effects of MP exposure in biota from aquatic environments, where MP abundance is most often reported [3,4,5,6,7,8].

The present study, therefore, investigated the toxic effects of MPs in *Tubifex tubifex*, which inhabits sediments of lakes and rivers. *T. tubifex* (also referred to as sludge or sewerage worm) lives in the uppermost sediment layers of these freshwater systems usually feeding in the sediment fraction smaller than 63 µm [23]. *T. tubifex* plays a key role in the decomposition of organic matter and bioturbation [24], dwelling in the sediment with the anterior part and keeping the tail outside, undulating it in the water to enable cutaneous respiration [6]. It is considered to be a model organism to perform sediment toxicity experiments [25,26] due to its high pollution tolerance [27] and is thus often one of the last species to disappear from a polluted habitat [24,28]. *Tubifex* worms are known to accumulate heavy metals [29,30,31] and organic pollutants [32] and are able to ingest MPs under natural conditions [33].

We, therefore, selected *T. tubifex* as a test subject to investigate the possible effects of MP pollution it may be exposed to in its environment. The aim of the present study was to understand whether exposure to MPs could affect the oxidative stress status of *T. tubifex*. *Tubifex* worms were exposed to fluorescent polyethylene (PE) MP particles (10 µm diameter) to evaluate the survival of the worms and the potential oxidative stress induced by MPs. PE was chosen because it is one of the most abundant polymers identified among samples collected from aqueous environments [7]. The size was selected within the size range of particles the worms could consume.

The enzyme activity of the oxidative stress biomarker enzymes GR and peroxidase (POD) were measured to check for alterations. Both enzymes, GR and POD, are known to be able to counteract the damages caused by ROS species to cells [34,35,36,37] and are thus induced in response to an increased oxidative stress status. GR plays a vital role in the antioxidant system [37,38], acting as a reductant in oxidation-reduction processes catalyzing the reduction of glutathione disulfide (GSSG) to glutathione (GSH) using NADPH as a cofactor. POD is known for playing a pivotal role in preventing H_2_O_2_ causing damage to DNA, proteins, and cell membranes [34,37] and has often been used in monitoring stress induced by contaminants [35,36].

## 2. Materials and Methods

*T. tubifex* was cultured in 1 L beakers in a synthetic medium at a constant temperature of 20 ± 1 °C and low light (18 μmol photons/m^2^s) with a photoperiod of 16 h light to 8 h dark (permanent continuous culture at the University of Helsinki) for several weeks before experimentation. The synthetic medium (artificial freshwater) was exchanged every three to five days, and it consisted of de-ionized water, CaCl_2_ [240 μg/L], KCl [6 μg/L], MgSO_4_.7H_2_O [123 μg/L], and NaHCO_3_ [55 μg/L]. The *T. tubifex* worms were fed with dry fish food (Sera, Mikropan, analytical constituents: crude protein 47.6%, crude fat 8.7%, crude fiber 3.3%, moisture 6.0%, crude ash 11.6%) daily.

### 2.1. Exposure to Microplastics

To establish the mortality of the worms in response to MP exposure, the *T. tubifex* worms were exposed to polyethylene MP particles (up to 10 µm in diameter) in four treatments; i.e., (1) *T. tubifex* in artificial freshwater containing 2 g/L MP (w/v) without sediment, (2) *T. tubifex* in artificial freshwater and sediment with the sediment containing 2 mg/g MP (w/w), (3) *T. tubifex* in artificial freshwater and sediment with the media spiked with 2 g/L MP (w/v), and (4) *T. tubifex* in artificial freshwater and sediment with both the media (2 g/L w/v MP) and the sediment (2 mg/g w/w MP) containing MP. The surviving worms were counted after 24 h, 48 h, and 120 h. For each experiment (mortality and enzyme assays, for all treatments), negative controls without MPs were conducted in replicates of five in parallel.

In order to examine the effects on the oxidative stress status, *T. tubifex* worms in sediment and artificial freshwater were exposed to the polyethylene MP particles (up to 10 µm in diameter) for 24 h in two different scenarios, i.e., MP-contaminated soil (2 mg/g w/w MP) vs. MP-contaminated media (2 g/L w/v MP). After 24 h, the treated and control worms were removed from the glass beakers and washed three times each with artificial freshwater, and the surviving worms counted.

In each experimental setup, five replicates of six adult *T. tubifex* worms per replicate were used. Each independent replicate was set up in a glass beaker containing either contaminated non-sterile sediments from Vesijärvi Lake (Lahti, Finland) or contaminated artificial freshwater or both as specified above.

### 2.2. Oxidative Stress Status: Enzyme Assays

After rinsing the worms with artificial freshwater, the enzymes were extracted using a shortened protocol modified from Pflugmacher [39]. *T. tubifex* worms were homogenized in 20 mM sodium phosphate buffer (pH 7.0) and stirred for 20 min followed by centrifugation at 9.000× *g* for 20 min. The supernatant (S-9 fraction) was collected and the enzyme activities were assayed immediately.

The activity of peroxidase (POD, EC 1.11.1.7) was analyzed spectrophotometrically using guaiacol as substrate [40], which is oxidized in the presence of hydrogen peroxide (H_2_O_2_) to octahydrotetraguaiacol. Changes in color as absorbance were measured at 436 nm over 3 min at 30 °C.

Glutathione reductase (GR, EC 1.8.1.7) activity was analyzed according to Schaedle and Bassham [41] following the glutathione disulfide-dependent NADPH oxidation at 340 nm for 3 min.

Each spectrophotometric enzyme assay was performed in triplicate per independent replicate (15 readings) using an Infinite 200 Pro plate reader (Tecan). The enzyme activity was normalized against protein content, which was determined according to Bradford [42]. Enzymatic activities are reported in nkat/mg protein, where 1 kat is the conversion rate of 1 mol of substrate per sec.

During all steps of the laboratory work, the possibility of self-contamination was taken into consideration and therefore fleece clothing or other personal items which could serve as a source of MP contamination were avoided [43].

### 2.3. Data Analysis

Statistical analysis was performed using IBM SPSS Statistics version 25 (2017). All data were checked for normality and homogeneity by Shapiro-Wilks and Levene’s test. A normal distribution of the data was analyzed using one-way analysis of variance (ANOVA) followed by Tukey’s test. For not normally distributed data, the Kruskal-Wallis test was employed to identify differences amongst the treatments, followed by the Mann-Whitney test if necessary. To assess whether survival/mortality changed with time, a repeated-measures ANOVA was performed. The results were considered significant at an alpha value of 0.05. All values are reported as mean ± standard error.

## 3. Results

### 3.1. Mortality

**Figure 1:**
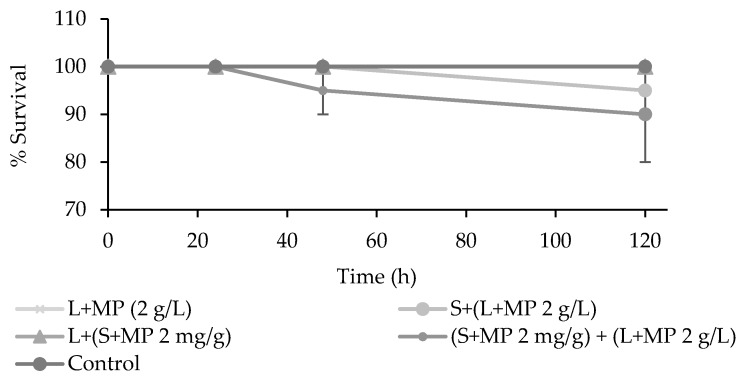
*Tubifex tubifex* mortality after exposure to microplastics (MPs) in media (L + MP (2 g/L)) only, in sediment and media with the media contaminated with MPs (S + (L + MP 2g/L), in sediment and media with the sediment contaminated (L + (S + MP 2 mg/g)), and sediment and media both contaminated with MPs ((S + MP 2 mg/g) + (L + MP 2 g/L)).

Contamination of the artificial water, sediment, or both water and sediment with MP (Figure 1) did not significantly affect the survival of *T. tubifex* in comparison to controls with time (*p* > 0.05). With contaminated artificial water and non-contaminated sediment, 95% ± 5% of the worms survived and with both sediment and water contaminated 90% ± 10% survived after five days. For all other treatments, 100% survived.

### 3.2. Oxidative Stress Status

**Figure 2:**
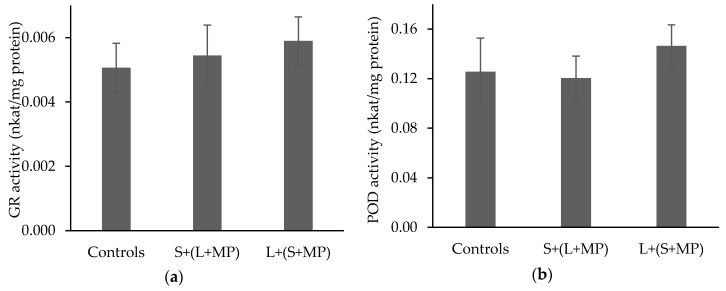
Peroxidase (**a**) and glutathione reductase (**b**) activities in *T. tubifex* exposed to fluorescent polyethylene (PE) microspheres through the media (S + (L + MP)) and the sediment (L + (S + MP). Values are expressed as mean enzyme activity ± standard error (n = 5). * denotes significance compared to the control (*p* < 0.05).

Exposure of *T. tubifex* to fluorescence PE microspheres did not result in significant (*p* > 0.05) changes in the GR activity in comparison to the controls (Figure 2a). The mean GR activities in the worms with MP contaminated water (S + (L + MP)) and contaminated sediment (L + (S + MP)) were 0.005 ± 0.001 and 0.006 ± 0.001 nkat/mg protein, respectively, compared to the control GR activity of 0.005 ± 0.001 nkat/mg protein.

Peroxidase activity with exposure of *T. tubifex* to fluorescence PE microspheres, as observed for GR, did not result in significant (*p* > 0.05) changes in POD activity in comparison to controls (Figure 2b). Mean POD activities with the water (S + (L + MP)) and sediment (L + (S + MP)) contaminated respectively, were 0.120 ± 0.002 nkat/mg protein and 0.146 ± 0.002 nkat/mg protein, respectively, and the control POD activity was 0.130 ± 0.003 nkat/mg protein.

## 4. Discussion

Oxidative stress has been investigated intensively in many organisms [44,45,46] and it has been shown to cause damages to DNA, lipids, and proteins, potentially leading to an alteration in vital functions [47,48,49,50]. However, studies regarding oxidative stress and the alterations in antioxidant enzyme activities due to exposure to MPs are still limited [20,21].

In this study, *T. tubifex* was exposed to fluorescent PE microspheres to evaluate the potential damages caused by MPs as percentage mortality or survival and on a physiological level as induced oxidative stress. Mortality percentage did not show any difference between treatment and control samples. These results are in accordance with Redondo-Hasselerharm et al. [51], where no effects on the mortality rate of five freshwater benthic macroinvertebrates (including a *Tubifex sp*.) were found after exposure to polystyrene MPs for 28 days.

No significant differences were evident in terms of GR activity of *T. tubifex* exposed to MP via contaminated water and sediment. Similarly, POD activity did not differ significantly in comparison to controls in both of the experiments. These data show that the selected MP concentrations are not particularly critical for *T. tubifex* and they do not cause oxidative stress to the worms. Nevertheless, our results do not imply that MPs are not representing a threat to the biodiversity of aquatic environments. Contrary to our findings, Jeong at al., [20] and Lu et al., [21] showed that exposure to PS MPs caused oxidative stress in the monogonont rotifer (*Brachionus koreanus*) and Zebrafish (*Danio rerio*), respectively. However, we have to take into consideration the different targeted organisms and the type and size of MPs used in the studies. Either the chosen test subjects may have been more susceptible than *T. tubifex* to MPs exposure, or the selection of a different polymer could explain the discrepancy between the results. Even though exposure to PE MPs seems not to increase the production of ROS in *T. tubifex*, it does not mean that MP occurrence is not able to negatively affect freshwater environments. *T. tubifex* is considered to be a pollution-tolerant species [24,28], and the effects of MPs could pose a major risk to more sensitive organisms [11,20,21]. Furthermore, the concentration of MPs is expected to increase worldwide [52] representing a possible threat to the biodiversity of marine and freshwater environments. Further research is required to understand the effects in more sensitive organisms. In this study, no estimate on how many PE MPs were ingested has been made; a follow-up study should be performed repeating the tests changing the level of MP exposure and performing fluorescence microscopy analyses to examine the MP ingestion rate.
